# Management of Recurrent Vaginitis

**DOI:** 10.1007/s13669-026-00494-w

**Published:** 2026-10-14

**Authors:** Anna M. Powell, Jack D. Sobel, Christina A. Muzny

**Affiliations:** 1 Department of Gynecology and Obstetrics, Johns Hopkins University School of Medicine, Baltimore, MD USA; 2 Division of Infectious Diseases, Department of Medicine, Wayne State University, Detroit, MI USA; 3 Division of Infectious Diseases, Department of Medicine, University of Alabama at Birmingham, Birmingham, AL USA

**Keywords:** Bacterial vaginosis, Vaginal candidiasis, *Trichomonas vaginalis*, Recurrent vaginitis

## Abstract

**Purpose of Review:**

This review summarizes current evidence regarding the pathogenesis, diagnosis, and management of recurrent BV, recurrent VVC, and persistent trichomoniasis while highlighting emerging therapies and research priorities.

**Recent Findings:**

Recurrent BV is driven by persistence of a polymicrobial biofilm on vaginal epithelial cells, failure to restore a *Lactobacillus*-dominant vaginal microbiome following antibiotic therapy, and reinfection from untreated sexual partners. Emerging therapeutic approaches including live biotherapeutic products, vaginal microbiota transplantation, and male partner treatment seek to address these mechanisms beyond conventional antibiotic suppression. In contrast, recurrent vulvovaginal candidiasis primarily reflects host susceptibility and immune dysregulation rather than persistent infection, making long-term suppressive antifungal therapy the cornerstone of management while newer antifungal agents expand treatment options for selected patients. Persistent trichomoniasis most commonly results from reinfection or incomplete therapy, although antimicrobial resistance remains an important consideration requiring specialized diagnostic testing and alternative treatment regimens.

**Summary:**

Recurrent vaginitis is a common and frustrating clinical problem that substantially impairs women’s quality of life and accounts for frequent healthcare utilization. Although bacterial vaginosis (BV), vulvovaginal candidiasis (VVC), and trichomoniasis (TV) share overlapping signs and symptoms, the mechanisms underlying recurrence differ considerably and require disease-specific management strategies.

## Introduction

Women with recurrent vaginitis symptoms present a common clinical challenge for healthcare providers. Generally, symptoms of vulvovaginitis account for some of the most frequent visits to a gynecologist’s office [1]. Recurrent vaginitis is associated with a negative quality of life and affected women often seek alternative treatments, such as oral probiotics or vaginal boric acid [2]. Accurate diagnosis of acute vulvovaginitis remains challenging despite the advent of newer molecular diagnostic tests, and many clinical settings still rely on empiric diagnosis and treatment to drive care [3]. In a recent randomized controlled trial, molecular diagnostic testing was found to significantly outperform the diagnostic accuracy of wet mount microcopy [4]. At baseline, 50–80% of women with bacterial vaginosis [BV] [5], 50% of women with yeast vaginitis [6] and 10–25% of women with trichomoniasis will experience a recurrence [7] following an initial episodic infection, though etiologies for recurrences vary. Also importantly, not all patients experiencing recurrent symptoms of vulvovaginitis (typically vaginal itching, discharge, and/or irritation) have an infectious etiology and non-infectious causes should also be considered. In a 2019 molecular diagnostic study, 35% of vaginal swabs were negative for all infectious targets (BV, VVC, trichomoniasis, and STIs) on comprehensive qPCR testing [8]. While molecular diagnostic testing plays an important role in vaginitis testing, wet prep microscopy is still recommended to evaluate alternative diagnoses, including inflammatory vaginitis conditions. In this review, we will provide updates on the management of recurrent vaginal infections with BV, yeast, and trichomoniasis.

### Recurrent Bacterial Vaginosis

Approximately one in three women will experience episodic BV within their lifetime, with an estimated recurrence rate of approximately 50–80% [5]. Current Centers for Disease Control Sexually Transmitted Diseases treatment guidelines [9] center on management of symptomatic syndromes with antibiotic therapies. Recurrent BV is defined as ≥ 3 episodes per year. The pathogenesis of recurrent BV is attributable to three overlapping drivers- persistence of *Gardnerella*-anchored polymicrobial bacterial biofilm, failure to re-establish *Lactobacillus*-dominant microbiota following antibiotic treatment, and reinfection/re-exposure from an untreated sexual partner [9–11].

#### Biofilm Persistence

Use of 5-nitroimidazoles (metronidazole [MTZ], tinidazole [TDZ], and secnidazole [SEC]) suppresses but does not fully eradicate the biofilm, facilitating re-emergence following completion of the antibiotic course [12]. In refractory cases, core biofilm species do not decline after oral metronidazole (MTZ), whereas these species declined in women who achieved BV remission [13, 14].

Other non-antibiotic approaches with anti-biofilm activity include dequalinium chloride and astodrimer sodium, although neither is currently available in the United States. Dequalinium chloride, a broad-spectrum antiseptic, disrupts established *Gardnerella* spp. biofilms and reduces biofilm biomass and metabolic activity in vitro [15]. Astodrimer sodium is a polyanionic dendrimer that blocks bacterial adhesion to vaginal epithelial cells and both inhibits biofilm formation and disrupts established biofilms. Clinical trials of astodrimer have demonstrated efficacy for both treatment and prevention of recurrent BV [16, 17].

#### Failure to Repopulate with Beneficial *Lactobacillus spp.*

5-Nitroimidazoles are bactericidal to anaerobic bacteria but do not restore protective, lactic-acid producing *L. crispatus *[18]. Following antibiotic treatment, anaerobic bacterial abundance significantly decreases but so does the overall bacterial load. Early recolonization typically occurs with *L. iners*, and is thought to outcompete *L. crispatus* growth [19, 20]. Two recent trials have shown some promise regarding the use of vaginal live biotherapeutic products (LBPs) to target this mechanism: Lactin-V and VIBRANT. In the Lactin-V trial, the intention-to-treat analysis showed that this investigational agent (a LBP containing a single *Lactobacillus* strain, *L. crispatus* CTV-05) significantly reduced BV recurrence through 24 weeks, but with attenuated durability compared to the primary 12-week outcome [21]. The VIBRANT study, a randomized phase 1b trial, evaluated two vaginal LBPs containing multiple *Lactobacillus* strains following standard antibiotic treatment for BV. In this study, participants were given placebo, 3, or 7 days of active LBP following multi-dose oral metronidazole treatment for BV. LBP strains were detected by metagenomics in 66.1% (47/71) of participants within the first 5 weeks, and half remained colonized at the 12-week endpoint [22].

#### Treatment of Recurrent BV

**Fig. 1:**
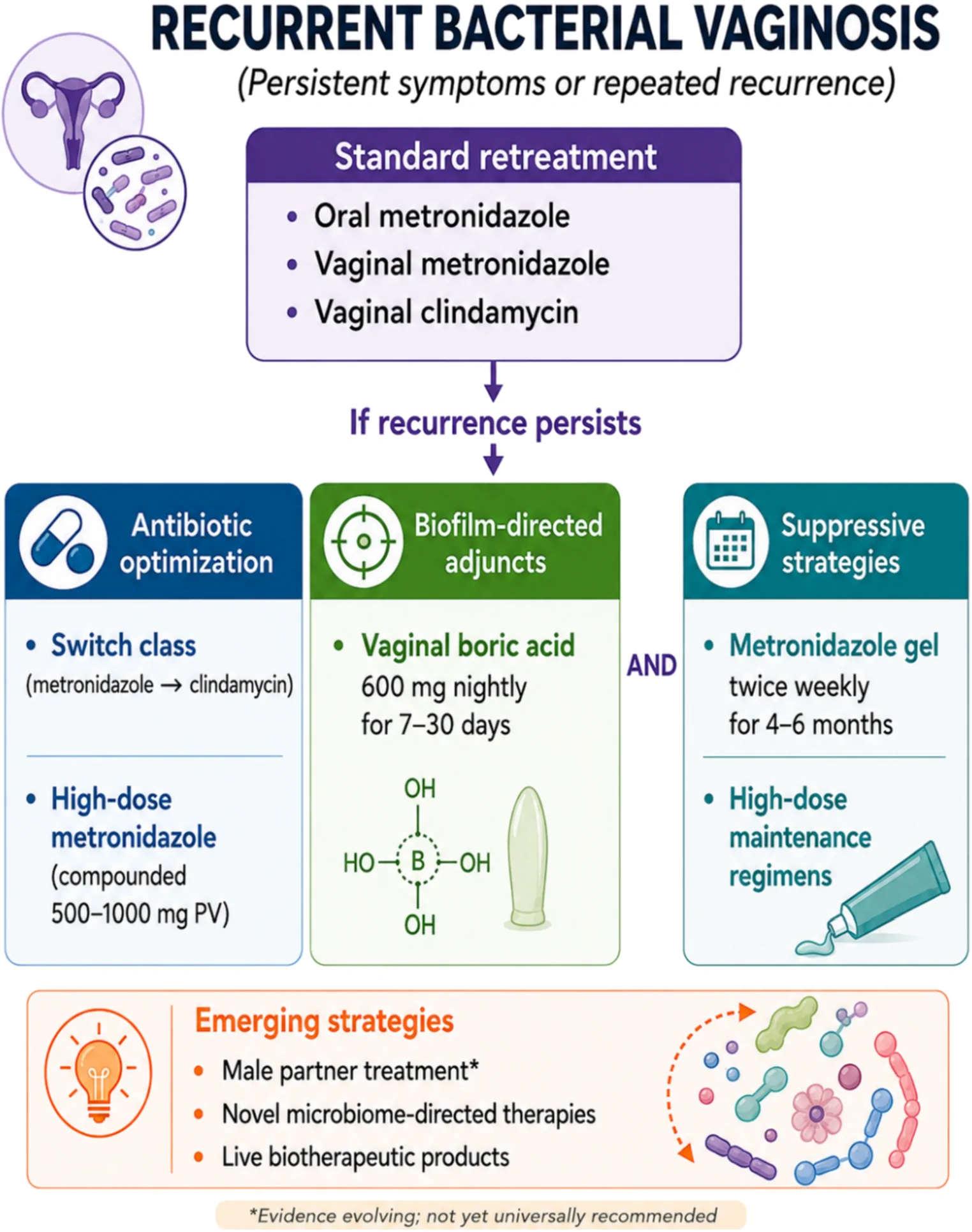
Recommended management of recurrent bacterial vaginosis (Adapted from Muzny and Sobel [23])

In the setting of an initial disease recurrence, a provider can switch to an alternative medication class (e.g. switch from oral metronidazole to vaginal clindamycin), followed by a 6-month course of twice-weekly vaginal metronidazole gel [23]. Further recurrence post-induction regimen failures may happen, in which case the provider can consider combination therapy with 21–30 days of intravaginal boric acid to the vaginal metronidazole maintenance regimen or intravaginal boric acid with use of high-dose compounded metronidazole [23, 24] (Fig. 1). Concurrently, other interventions should include adherence to condom use with male partners [25], as well as use of hormonal contraception as each of these factors may modify BV susceptibility [26, 27].

Other lines of investigation towards the goal of repopulating favorable strains of *Lactobacillus spp.* include studies of vaginal microbial transplant (VMT) [28–30]. VMTs remain highly experimental, though conceptually they attempt to accomplish what standard antibiotic treatment alone cannot: eradicate dysbiosis with antibiotic pretreatment, replace the entire vaginal microbial community, and prevent recolonization with BV-associated bacteria (BVAB). The first such trial was conducted by Lev-Sagie et al. [28] in patients with recurrent BV; 4 of 5 study participants experienced complete clinical remission. Remission was characterized by sustained *Lactobacillus spp.* dominance, disappearance of BVAB, normalization of Nugent scores and symptom resolution, without any serious treatment-related adverse events reported [28].

#### Male Partner Treatment of BV

Emerging interventional data supports sexual transmission of BVAB from male to female sexual partners resulting in incident BV [10, 31–33]. In March 2025, the Australian StepUp trial demonstrated a significant reduction in subsequent BV episodes following regular male sexual partner treatment (MPT) with combination antibiotic therapy (multi-dose oral metronidazole and 2% topical clindamycin cream applied to the penis twice daily X 7 days) compared to standard of care (female partner only treatment). Notably, women with a lifetime history of BV were included but the trial did not specifically evaluate women with recurrent BV, defined as ≥ 3 episodes per year. Recurrent BV is considered an inherently different condition from sporadic BV, as recurrent BV is typically attributed to an established/adherent bacterial community (biofilm), considered to be more resilient against antibiotics [23]. Several pros and cons of BV MPT are outlined in Table 1. BV MPT has been recently endorsed for consideration by the American College of Obstetrics and Gynecology (ACOG) [34] and International Society for the Study of Vulvovaginal Disease (ISSVD) [35], though providers should note the above caveats in extrapolating StepUp trial data to women with recurrent BV, and consider offering MPT through shared decision making to motivated monogamous couples after an initial BV episode (as per inclusion criteria in StepUp).

**Table 1:** Pros and cons of BV male partner treatment

	PRO	CON	Comment
Acceptability of MPT	MPT was widely acceptable to study participants when BV was reframed as a shared responsibility between partners [36]	Acceptability for MPT in the general population may vary widely outside of the motivated group of study participants within the StepUp trial	
Generalizability of StepUP findings	Included women with and without a prior history of BV makes the trial highly inclusive of a population most likely to be encountered in general clinical practice	Few Black women or women of African ancestry included.Concern for insufficient data to extrapolate StepUp findings to US-based male populations (20% [*n* = 14] of circumcised study participants)	
Adherence of male partners	Higher adherence with medications within the trial resulted in lower female partner BV recurrence rates	Real world adherence may be lower	Condom use adherence following therapy is also critical
Trial length of duration	A significant decrease in BV recurrence rates seen within 12 weeks	Unknown benefit past 12 weeks	This critique assumes no other treatments will be administered; MPT will likely be part of a larger maintenance treatment strategy and not a stand- alone intervention
Antimicrobial stewardship	The number needed to treat within StepUp was 4 couples, suggesting a significant benefit to administering MPT to the right couple after shared decision making	Concern for additional antibiotic doses for “unproven benefit”	Providers should counsel couples about risks/benefits/alternatives to using MPT and employ shared decision making The current standard of care subjects women with recurrent BV to prolonged antibiotic exposures that could be curtailed by BV MPT

### Management of Recurrent Vulvovaginal Candidiasis (RVVC)

RVVC is defined as ≥ 3 episodes of symptomatic VVC in the previous year. According to Denning et al. [37], RVVC affects over 138 million women annually with highest rates in 25-24-year-olds. By 2030, the RVVC population is estimated to reach ~ 158 million individuals. It follows that the economic burden annually is extremely high, as are the emotional and physical consequences [38]. However not all women with RVVC are equal and may be classified as primary (idiopathic, often familial) or secondary RVVC with different pathophysiologic underlying mechanisms and treatment approaches. Idiopathic RVVC almost always includes azole-sensitive *Candida albicans* isolates, with individual episodes of VVC being unaccompanied by obvious causal factors or “triggers” responsible for individual episodes (Table 2). Management of RVVC will vary according to these contributory mechanisms. For the rest of this section, treatment will focus on the extremely common idiopathic RVVC almost always caused by *C. albicans*.

**Table 2:** Triggers for sporadic episodes of VVC [9]

Exposure	Example
Systemic antibiotics	Secondary yeast infection following BV treatment
Estrogen elevation	Pregnancy, diabetes mellitus (and SGLT-2 inhibitors; e.g., empagliflozun), exposure to estrogen-containing medications (e.g., hormonal contraceptives and hormone treatment during menopause)
Immunosuppression	HIV, systemic corticosteroid use, cancer

SGLT-2 = sodium glucose co-transporter 2

The primary initiating step in VVC involves vaginal colonization with *Candida* species originating in gastrointestinal and vaginal reservoirs, which are uncommonly sexually transferred [39]. These reservoirs can lead to asymptomatic colonization or proliferate and express well-known yeast virulence factors initiating vaginal mucosal invasion. Vaginal colonization with *Candida* spp. is common and does not invariably result in symptomatic disease. Susceptibility to idiopathic RVVC appears to be influenced, at least in part, by host genetic variation, including polymorphisms affecting innate immune recognition and mucosal inflammatory responses to *Candida* spp. Thus, host genetics may influence whether vaginal *Candida* spp. colonization is tolerated asymptomatically or results in an exaggerated inflammatory response and symptomatic VVC [40, 41].

The pathogenesis of RVVC differs between idiopathic and secondary/recurrent disease. Secondary RVVC occurs in the setting of identifiable host or environmental factors that promote *Candida* spp. colonization, proliferation, or impaired mucosal defense, including systemic antibiotic exposure, elevated estrogen states, diabetes mellitus, sodium-glucose cotransporter-2 inhibitor use, corticosteroid therapy, and other forms of immunosuppression. In these patients, management should address the underlying predisposing factor when possible.

In contrast, women with idiopathic RVVC generally lack an identifiable external trigger and typically have azole-susceptible *C. albicans*. Increasing evidence suggests that susceptibility in these women reflects differences in the host response to *Candida* spp. Host genetic polymorphisms affecting innate immune recognition and mucosal inflammatory pathways have been associated with RVVC susceptibility [42]. In susceptible women, proliferation and mucosal invasion by *Candida* may provoke an exaggerated local inflammatory response, resulting in symptomatic vulvovaginitis [43]. Accordingly, idiopathic RVVC may be conceptualized as a disorder of host–pathogen interaction in which the clinical phenotype depends not only on the presence of *Candida* spp., but also on the host inflammatory response to the organism [44]. Previous theories that RVVC is caused by non-protective VMB or hypervirulent VVC strains have not been substantiated. This host-centered model is also consistent with the limited evidence that manipulation of the vaginal microbiota with commercially available probiotics prevents RVVC [45] and with the failure of strategies aimed at suppressing the gastrointestinal *Candida* spp. reservoir to prevent recurrence [6, 46].

An important distinction in idiopathic RVVC is that successful treatment of an acute episode should not be equated with durable cure of the recurrent disease phenotype. Antifungal therapy can produce both clinical resolution and negative post-treatment cultures, demonstrating effective clearance or suppression of vaginal *Candida* spp. Maintenance antifungal therapy likewise markedly reduces symptomatic recurrences while treatment is continued. However, around 50% of women with idiopathic RVVC experience early recolonization and recurrent symptomatic disease after maintenance antifungal therapy is discontinued [6, 47]. Current therapies generally do not eliminate the underlying host susceptibility that predisposes some women to repeated episodes of symptomatic VVC.

Genetic polymorphisms also contribute to host vaginal mucosal proinflammatory defense mechanisms that influence “tolerance” to yeast presence in the vaginal microbiome. Women with idiopathic RVVC have evidence of an enhanced mucosal inflammatory reaction with clinical manifestations of vulvovaginitis reflecting “intolerance” to proliferating and invading yeast microorganisms. In contrast, secondary RVVC occurs in the setting of identifiable factors that promote *Candida* colonization or proliferation or impair local host defenses, including SGLT-2 inhibitor use, elevated estrogen states, and corticosteroid therapy. Non-*albicans Candida* spp. represent a distinct clinical challenge because of their reduced susceptibility or intrinsic resistance to commonly used azole antifungals. Fortunately to date, azole resistant *C. albicans* isolates uncommonly cause RVVC [6, 48, 49]. Attempts to reduce RVVC by suppressing the gastrointestinal yeast reservoir have invariably failed. In contrast, use of systemic maintenance antifungal oral agents that suppress or reduce vaginal yeast colonization have been highly effective, even long term, but only as long as prophylactic antifungals are used, with high rates of recolonization and symptomatic RVVC following drug cessation but associated with continued azole drug susceptibility [50, 51].

#### Clinical Aspects of RVVC

Clinical expression of RVVC attacks is highly variable with mild to severe signs of vulvovaginal inflammation, not different from sporadic episodes. RVVC may be “chronic” due to incomplete mucosal control and present as secondary dyspareunia only.

#### Treatment of Idiopathic RVVC

For many years practitioners resorted to treating multiple individual episodes of RVVC without maintenance prophylactic regimens. This ended with the availability and convenience of newer oral azole drugs. Multiple randomized controlled trials have verified the safety and efficacy of fluconazole and a variety of dosing regimens now exist, each with marginal advantage only [52, 53]. All prophylactic oral azole “regimens” share common features, but are to be avoided during pregnancy, achieving symptom free lifestyles but are nevertheless not curative [50, 51, 54], changing the lives of millions of women worldwide with rare occurrence of drug resistance and few adverse effects. Similar benefit can be obtained with topical azole agents administered daily and occasionally once weekly, but especially useful during pregnancy [55, 56].

Recently, ibrexafungerp, a non-azole antifungal, was approved by the U.S. FDA for RVVC [57]. However, the drug is not currently commercially available. From an efficacy perspective, this agent has no advantage over once weekly fluconazole except in women allergic, intolerant, or resistant to fluconazole. A new highly potent oral azole, oteseconazole, prescribed once weekly for 12 weeks is now available with an extremely prolonged half-life resulting in continued RVVC prophylactic efficacy for 1–2 years following drug cessation [58]. However, U.S. FDA directives preclude use of this more convenient potentially more effective antifungal drug in women with reproductive capacity including use in pregnancy, markedly reducing its availability. It follows that pregnant women with RVVC should be prescribed daily only or weekly topical azoles or intravaginal nystatin for the duration of pregnancy.

#### Treatment of Secondary RVVC (*C. albicans*)

Management of this group of women employs the same principle of long-term maintenance azole drug (fluconazole) prophylaxis but also targets and reverses causal factors. This includes cessation of drugs where possible (e.g., steroids, estrogen, control of diabetes, and BV). Failure to eliminate those factors is unfortunately frequent and requires addition of effective weekly fluconazole. One cannot control RVVC in women with recurrent BV without controlling the BV process.

#### Treatment of Azole-Resistant RVVC (*C. albicans*)

Symptomatic RVVC due to azole-resistant *C. albicans* is now more common and is best treated with ibrexafungerp when available. Antifungal therapy may achieve clinical and mycologic control of the current episode, but recurrence following treatment remains common. All cases with clinical suspicion of azole resistance should be confirmed by antifungal susceptibility tests, which unfortunately are not widely available and rarely provide active alternative drugs. In the absence of such information, a trial of nightly intravaginal boric acid 600 mg suppositories for at least 14–21 days or daily topical nystatin 100,000 units should be attempted [59]. Recognizing that while temporary relief is likely, that as with fluconazole, cure is unlikely. Accordingly, following an initial favorable response to these agents, long-term maintenance daily and then intermittent thrice weekly can be prescribed long-term.

#### Treatment of Non-Albicans Candida Species

Most of these non-*albicans Candida* spp. are less virulent and serve as commensals only. Hence when isolated in women with RVVC, one is required to confirm causality. It is essential to establish that such isolates are responsible for recurrent or chronic symptoms. Because of the frequent azole drug resistance associated with these infrequent pathogens, referral to specialist practitioner is advised or initial use of boric acid, nystatin, topical amphotericin B, flucytosine alone or in combination is recommended [60].

Finally, use of commercially available probiotics or male partner treatment is not advised in women with RVVC [60]. However new approaches are being explored including anti-*Candida* spp. vaccines which aim at cure as well as control of RVVC [6, 61]. Studies are also in progress evaluating vaginal zinc supplements [60]. Unfortunately, genetic tests are not available to diagnose sporadic VVC and RVVC susceptibility.

## Management of Recurrent/Persistent Trichomoniasis

Persistent trichomoniasis is defined as a *Trichomonas vaginalis* infection that fails to clear despite standard therapy. When evaluating persistent cases, clinicians should categorize the cause into one of four categories: incomplete medication adherence to a standard *T. vaginalis* treatment regimen, reinfection from an untreated sexual partner, infection from a new partner, or true refractory infection due to drug resistance [62]. Currently, oral 5-nitroimidazoles (MTZ, TDZ, and SEC) are the only class of medications approved by the U.S. FDA for *T. vaginalis* treatment [62]. MTZ, a first generation 5-nitroimidazole, was first FDA-approved for *T. vaginalis* treatment in 1959 [63, 64]; it has a half-life of 7–8 h. TDZ, a second generation 5-nitroimidazole with a half-life of 12–13 h, was subsequently FDA-approved for *T. vaginalis* treatment in 2004 [65]. SEC, another second generation 5-nitroimidazole with a longer half-life of 17–19 h, was FDA-approved in 2021 [66]. MTZ, TDZ, and SEC share a common imidazole ring structure with a methyl group at position 2 and a nitro group at position 5. Their chemical structure varies based on the side chains attached to a nitrogen group at position 1 of the imidazole ring structure [67].

### Current Recommended and Alternative *T. vaginalis* Treatment Regimens

Current recommended and alternative *T. vaginalis* treatment regimens for MTZ and TDZ, based on the 2021 CDC STI Treatment Guidelines, are listed in Table 3 [9]. Of note, a single 2-gram dose of oral SEC for *T. vaginalis*-infected women and men was FDA-approved after these guidelines were written. However, single dose oral SEC has since been recommended as an alternative treatment option for *T. vaginalis*-infected women and men by the World Health Organization (WHO) [68].

**Table 3:** Current recommended and alternative treatment regimens for *T. vaginalis* [9, 68]

Regimen	Duration of therapy
Recommended	
Oral MTZ 500 mg twice daily (women) Oral MTZ 2 g single dose (men)	7 days Single dose
Alternative	
Oral TDZ 2 g single dose (women and men) Oral Sect. 2 g single dose (women and men)	Single dose Single dose

MTZ metronidazole, TDZ tinidazole, SEC secnidazole

### Prevalence of Drug-Resistant *T. vaginalis* Infections

The prevalence of 5-nitroimidazole drug resistant *T. vaginalis* infection is not known, as *T. vaginalis* is not a nationally reportable disease [69, 70]. Data on the prevalence of drug resistance are thus only available from small studies which suggest that it occurs in approximately 4–10% of *T. vaginalis* infections [71, 72]. Complicating this picture is that 5-nitroimidazole drug resistance in *T. vaginalis* infection may be relative and not absolute [72, 73].

### Pathogenesis of Drug-Resistant *T. vaginalis* Infection

Mechanisms of 5-nitroimidazole drug resistance is an area of active research. Previous studies have linked drug resistance to altered hydrogenosomal metabolism in the *T. vaginalis* parasites, impaired 5-nitroimidazole activation, and shifts in cellular redox balance [74, 75]. A recent study of 15 MTZ-sensitive *T. vaginalis* isolates and 15 MTZ-resistant isolates observed extensive cross-resistance among MTZ, TDZ, and SEC, suggesting that drug resistance mechanisms are conserved across the 5-nitroimidazole class. This study found that iron (an essential component of hydrogenosomal metabolism) import restriction was a central feature of *T. vaginalis* drug resistance. This finding supports a model in which resistant *T. vaginalis* isolates restrict iron uptake at the cell-membrane level, limiting intracellular iron availability and reducing 5-nitroimidazole activation within the hydrogenosome [67]. Additional studies with larger number *T. vaginalis* MTZ-sensitive and resistance isolates need to be conducted.

### Management of Refractory *T. vaginalis* Infection

The management of refractory *T. vaginalis* infection in the setting of 5-nitroimidazole drug resistance is a significant clinical challenge given that there are no standardized, highly effective second-line treatment options. If medication non-adherence, reinfection, and new infection have been ruled out and drug resistance is suspected, *T. vaginalis* culture and 5-nitroimidazole susceptibility testing should be performed [62]. This testing is currently available for MTZ and TDZ at the CDC Division of Parasitic Diseases and Malaria; a kit may be requested at https://www.cdc.gov/laboratory/specimen-submission/detail.html?CDCTestCode=CDC-10239. Additionally, testing for MTZ, TDZ, and SEC drug resistance is also available at the University of Alabama at Birmingham (UAB) Sexually Transmitted Infectious Research Laboratory in special circumstances (call 205-975-5665 for further information).

While waiting on 5-nitroimdazole drug susceptibility testing results and given that *T. vaginalis* drug resistance may be relative and not absolute, treatment with higher doses of 5-nitroimidazoles may be able to overcome the resistance. In this setting, 2 g of oral MTZ or TDZ daily for 7 days should be initially given. If the patient fails this regimen, combination oral and intravaginal therapy is recommended. Several regimens are listed in Table 4. In settings where combination therapy fails, use of intravaginal boric acid 600 mg twice daily for 60–90 days should be considered [76]. Repeat *T. vaginalis* nucleic acid amplification testing (NAAT) to monitor for resolution of infection should not be obtained until at least 3 weeks after the end of treatment [77].

**Table 4:** Combination therapy regimens in the setting of refractory *T. vaginalis* infection

Regimen*	Duration of therapy
Oral TDZ 2–3 g daily in divided doses + Intravaginal TDZ 500 mg twice daily [78]	14 days
Oral TDZ 2–3 g daily in divided doses + Intravaginal paromomycin^€^ 4 g of 6.25% cream nightly [79]	14 days
Oral Sect. 2 g daily + Intravaginal boric acid^µ^ 600 mg twice daily [80]	14 days

*Intravaginal medications have to be prepared at a compounding pharmacy

^€^Common local side effects of vaginal paromomycin include vaginal irritation, itching, redness, or dryness. Localized vaginal ulcerations may also occur, which generally resolve on their own after treatment discontinuation

^µ^Common local side effects of vaginal boric acid include a mild burning sensation, watery discharge, and local skin irritation; these effects are usually mild and temporary

### Novel Drug Classes for Treatment of Refractory *T. vaginalis* Infection

Research of novel drug classes for treatment of refractory *T. vaginalis* infection is ongoing. These have included agents within the thiazolide drug class, nitazoxanide and tizoxanide, in addition to a derivative of nitazoxanide, amixicile [81]. Additionally, the in vitro activity of a patent-pending novel ozonated oil (RD011926) against *T. vaginalis* has also recently been demonstrated in a small study [82, 83].

## Conclusion

Recurrent vaginitis remains one of the most common yet challenging conditions encountered in gynecologic practice. Although recurrent BV, RVVC, and persistent trichomoniasis often present with similar signs and symptoms, their underlying biologic mechanisms differ substantially, underscoring the importance of accurate diagnosis and individualized management rather than empiric retreatment alone. Recent advances have shifted the field beyond repeated antimicrobial/antifungal courses toward mechanism-based interventions, including vaginal microbiome restoration, disruption of bacterial biofilms, optimization of host immunity, and interruption of sexual transmission. While several promising therapies—including live biotherapeutic products, vaginal microbiota transplantation, male partner treatment for BV, novel antifungal agents, and alternative therapies for drug-resistant *T. vaginalis*—have demonstrated encouraging early results, additional randomized controlled trials are needed to establish long-term efficacy, durability, safety, and optimal patient selection. Future therapeutic strategies will likely combine antimicrobial treatment with microbiome-directed and host-directed interventions to achieve sustained remission rather than temporary suppression of disease.

## Key References

- Vodstrcil LA, Plummer EL, Fairley CK, et al. Male-Partner Treatment to Prevent Recurrence of Bacterial Vaginosis. N Engl J Med. 2025;392:947–957.
  - ○ This randomized StepUp trial demonstrated that treating male partners with oral metronidazole plus topical penile clindamycin significantly reduced BV recurrence compared with treating women alone, providing compelling interventional evidence that sexual exchange of BV-associated bacteria contributes to recurrence. The study represents an important shift toward addressing reinfection from sexual partners as a modifiable driver of BV recurrence, although the population was not specifically restricted to women meeting a recurrent-BV definition.
- Kissinger PJ, Gaydos CA, Seña AC, et al. Diagnosis and Management of *Trichomonas vaginalis*: Summary of Evidence Reviewed for the 2021 CDC STI Treatment Guidelines. Clin Infect Dis. 2022;74(Suppl 2):S152–S161.
  - ○ This evidence review provides the foundation for contemporary management of persistent *T. vaginalis*, emphasizing the need to distinguish nonadherence and partner reinfection from true antimicrobial resistance before escalating therapy. It also supports multidose 5-nitroimidazole treatment and susceptibility-guided approaches for refractory infection, which form the clinical framework used in your review.
- Lederman SN, Sobel R, Sobel JD, Curelop SB, Brand SR. Sustained Efficacy of Oteseconazole in Women with Recurrent Vulvovaginal Candidiasis. J Womens Health (Larchmt). 2026;35(3):334–339.
  - ○ Key finding: Following a 12-week course of oteseconazole, the protective effect against RVVC was remarkably durable: only 1 of 70 women (1.4%) experienced a culture-confirmed recurrence during weeks 48–96, despite receiving no additional study treatment during the extension period. This suggests that oteseconazole may provide sustained suppression well beyond treatment completion, although its clinical applicability is limited by its restriction to women who are not of reproductive potential.

## Data Availability

No datasets were generated or analysed during the current study.
